# Glycine Betaine Relieves Lead-Induced Hepatic and Renal Toxicity in Albino Rats

**DOI:** 10.3390/toxics10050271

**Published:** 2022-05-23

**Authors:** Farid Abdelrazek, Dawlat A. Salama, Afaf Alharthi, Saeed A. Asiri, Dina M. Khodeer, Moath M. Qarmush, Maysa A. Mobasher, Mervat Ibrahim

**Affiliations:** 1 Biochemistry Department, Faculty of Agriculture, Ain Shams University, Cairo 11566, Egypt; farid_abdelrazek@agr.asu.edu.eg (F.A.); dawlat_salama@agr.asu.edu.eg (D.A.S.); 2 Department of Clinical Laboratory Sciences, College of Applied Medical Sciences, Taif University, Taif 21944, Saudi Arabia; a.awwadh@tu.edu.sa; 3 Department of Clinical Laboratory Sciences, College of Applied Medical Sciences, Najran University, Najran 61441, Saudi Arabia; saaasiri@nu.edu.sa; 4 Department of Pharmacology & Toxicology, Faculty of Pharmacy, Suez Canal University, Ismailia 41522, Egypt; 5 Urology Department, Prince Sultan Military Medical City, Riyadh 12233, Saudi Arabia; mqarmush@psmmc.med.sa; 6 Department of Pathology, Biochemistry Division, College of Medicine, Jouf University, Sakaka 41412, Saudi Arabia; mmobasher@ju.edu.sa

**Keywords:** lead, glycine betaine, antioxidant enzymes, liver function, kidney function, lipid peroxidation, reduced glutathione

## Abstract

Lead (Pb) is a widespread and nondegradable environmental pollutant and affects several organs through oxidative mechanisms. This study was conducted to investigate the antioxidant protective effect of glycine betaine (GB) against Pb-induced renal and hepatic injury. Male albino rats (*n* = 45) were divided into three groups: G1 untreated control, G2 Pb-acetate (50 mg/kg/day), and G3 Pb-acetate (50 mg/kg/day) plus GB (250 mg/kg/day) administered for 6 weeks. For G3, Pb-acetate was administered first and followed by GB at least 4 h after. Pb-acetate treatment (G2) resulted in a significant decrease in renal function, including elevated creatinine and urea levels by 17.4% and 23.7%, respectively, and nonsignificant changes in serum uric acid levels. Serum aspartate aminotransferase (AST), alanine aminotransferase (ALT), and alkaline phosphates (ALP) activities were significantly increased with Pb treatment by 37.6%, 59.3%, and 55.1%, respectively. Lipid peroxidation level was significantly increased by 7.8 times after 6 weeks of Pb-acetate treatment. The level of reduced glutathione (GSH-R) significantly declined after Pb-acetate treatment. Pb-acetate treatment also reduced the activities of superoxide dismutase (SOD), glutathione-S-transferase (GST), and glutathione peroxidase (GSH-PX) by 74.1%, 85.0%, and 40.8%, respectively. Treatment of Pb-intoxicated rats with GB resulted in a significant reduction in creatinine, urea, ALT, AST, and lipid peroxidation, as well as a significant increase in the level of GSH-R and in the activities of ALP, SOD, GST, and GSH-PX. The molecular interaction between GB and GSH-PX indicated that the activation of GSH-PX in Pb-intoxicated rats was not the result of GB binding to the catalytic site of GSH-PX. The affinity of GB to bind to the catalytic site of GSH-PX is lower than that of H_2_O_2_. Thus, GB significantly mitigates Pb-induced renal and liver injury through the activation of antioxidant enzymes and the prevention of Pb-induced oxidative damage in the kidney and liver.

## 1. Introduction

Lead (Pb) is one of the major environmental and nondegradable pollutants, and overexposure to it results in several physiological and biochemical changes associated with liver and renal dysfunction [1]. Pb exposure causes renal toxicity and hepatotoxicity through its ability to induce oxidative stress, resulting in an imbalance between the production of reactive oxygen species (ROS) and the potency of antioxidant defense enzymes [2]. Pb exposure promotes ROS production that attacks biologically essential molecules, such as membrane lipids, proteins, DNA, and enzymes, and results in renal and hepatic dysfunction [2,3]. The generation of ROS due to exposure to Pb ions is known to damage several biological compounds, including nucleic acids, proteins, and cell membrane lipids [4]. The oxidative damage of biological macromolecules induced by Pb exposure has suggested a mechanism in which Pb-induced oxidative stress contributes to renal and hepatic Pb toxicity caused by disturbances in the balance between ROS generation and antioxidant activities in both hepatic and renal cells [5,6]. Moreover, some in vitro treatments of cells with Pb increased the production of ROS [7,8]. In vivo studies also confirmed that Pb treatments increased the production of ROS and caused remarkable changes in the activities of antioxidant defense systems in experimental animals [9,10,11,12]. Furthermore, Pb exposure causes alterations in antioxidant activities by inhibiting functional SH groups in several enzymes such as δ-aminolevulinic dehydrogenase (ALAD), superoxide dismutase (SOD), catalase (CAT), and glutathione peroxidase (GSH-Px) [10,13,14]. Glutathione plays a vital role in the scavenging of reactive oxygen radicals. The treatment of animals with Pb oxidized both GSH and GSSG enzymes and reduced the GSH/GSSG ratio, which acts as a good marker of oxidative stress [15]. The levels of reduced glutathione, GSH, in fish treated with Pb were decreased by the binding of Pb to SH groups in GSH, which affects the antioxidant activity of GSH [16].

Glycine betaine (GB; *N*,*N*,*N*-trimethyl glycine) is a natural compound isolated for the first time from sugar beets. It exists in many plant species. GB acts as an osmolyte in bacteria and some plant species [17]. The presence of GB was also detected in animals [18]. GB synthesis is catalyzed by enzymes such as choline dehydrogenase and betaine aldehyde dehydrogenase, which are present in both plants and animals. Several foods contain high GB amounts, such as spinach, beetroot, and wheat bran [18,19]. GB is an important metabolite for osmoregulation and for protection against metabolic disorders [20,21,22]. GB reduces membrane-lipid peroxidation and increases the activities of antioxidant enzymes, such as SOD, GSH-PX, and CAT. Moreover, GB induces the activities of GSH-S-transferase (GST) and paraoxonase 1 [23,24]. Furthermore, in rats with hepatic injury, GB increases the level of reduced glutathione and the activities of glutathione reductase (GR), glutathione peroxidase (GSH-PX), and glutathione S transfer activity (GSH-T) [25]. The ability of GB to increase the reduction in glutathione and the activities of antioxidant defense enzymes suggests that GB protects liver, kidney, and other organs against oxidative damage. There is extensive evidence suggesting that GB protects the liver and kidney from several disorders, such as fatty liver disease and alcoholic liver disease. Several studies indicated that gender is a critical factor in hepatotoxicity. Males were more susceptible to toxic liver substances than females. Estrogen concentration is the main reason for the gendered difference in responses to the hepatic toxicity [26,27]. Women’s higher tolerance for hepatic toxins could be attributed to the antioxidant potency of the female hormone estrogen [28]. To avoid the hormonal effect of estrogen on Pb-induced toxicity, only male rats were used in the present investigation.

This study was conducted to investigate the efficiency of GB in mitigating Pb-induced renal and hepatic injury and in improving the activities of antioxidant defense enzymes. The effects of GB on the hepatic enzymes, renal function, lipid peroxidation, reduced glutathione, and antioxidant defensive enzymes were highlighted. Furthermore, the molecular interaction between GB and GSH-PX was investigated.

## 2. Materials and Methods

### 2.1. Chemicals

TGB (*N*,*N*,*N*-trimethyl glycine), lead acetate, and thiobarbituric acid were purchased from sigma company in Egypt. Kits for determination of ALT, AST, ALP, creatinine, urea, and uric acid were provided by EgyChem for Lab Technology, Cairo, Egypt. The kit for the determination of reduced glutathione and the kits for the determination of the activities of GSH-PX, GSH-T, and SOD were provided by Egyptian Company for Biotechnology, Cairo, Egypt.

### 2.2. Animals and Experiment Design

The experimental design was carried out according to the precedent set by Jegede et al., 2015 [29], but with a necessary modification. A total of 45 male albino rats of Wistar strain with similar weight (235 g ± 5 g) were obtained from Animal Health Institute Dokki, Giza, Egypt. The animals were clinically checked and maintained on basal diets and under normal health conditions for a week to adapt to the laboratory conditions before starting the treatments. Rats were divided into three groups, with each group containing 15 rats, and the experimental diagnosis was carried out as follows:

**The control (G1):** rats received basal diet and drinking water.
**Pb-acetate treated(G2):** rats have ingested Pb-acetate in drinking water daily with a dose of 50 mg/kg bw daily for 6 weeks.
**Pb-acetate and GB-treated (G3)**: rats received Pb-acetate at 50 mg/kg bw followed by GB at 250 mg/kg bw after 4 h in drinking water daily for 6 weeks.

The animals received humane care and were handled according to the guidelines of the ethics committee of Ain Shams University.

After 6 weeks of treatment, blood samples were collected from retro-orbital vein with heparinized capillary tubes.

### 2.3. Serum Samples Preparation

The collected blood samples were kept at 4 °C for 30 min and then centrifuged at 3000× *g* for 15 min, and serum was collected and used later to determine the level of lipid peroxidation, the measurement of GSH-R, liver function, renal function, and antioxidant-defense enzymes’ activities.

### 2.4. Quantitative Determination of Lipid Peroxidation

The level of lipid peroxidation damage caused by free radicals in the cells was determined by measuring malondialdehyde (MDA) concentrations in serum samples with a spectrophotometer according to the method described by [30]. Serum samples (250 μL) were added to 650 μL of 5% trichloroacetic acid and 100 μL 0f butylated hydroxy methyl benzene (500 mg/L) dissolved in methanol. The mixtures were heated in a boiling water bath for 30 min. Then, the mixtures were centrifuged at 10,000× *g*. The supernatants were collected and added to the same volume of a saturated solution of 2-thiobarbituric acid. The absorbance of the colored supernatants was measured spectrophotometrically at 532 nm. The concentration of MDA was calculated with the extinction coefficient of 155 μM^−1^·cm^−1^ and expressed as nmole/dL serum sample.

### 2.5. Determination of GSH-R Levels

GSH-R levels were determined in the obtained serum samples using the colorimetric method described by [31]. Serum samples (250 μL) were added to 200 μL of cold distilled water (4 °C) and 500 μL of TCA 50% *w*/*v*; 4 °C. The mixture was centrifuged at g for 15 min, and then, 1 mL of the supernatant was collected and added to 2 mL of Tris buffer 40 mM, pH = 8.9, and 50 μL of 10 mM of 5,5′-dithio-bis (2-nitrobenzoic acid (DTNB)) in methanol. The absorbance was recorded at 412 nm. GSH-R concentration was calculated and expressed as mg/dL serum.

### 2.6. Serum Biochemistry

#### 2.6.1. Liver Function

Alanin aminotransferase (EC 2.6.1.2), ALT, and aspartate aminotransferase (AST) activities were determined in the serum samples according to the method described by Reitman et al., 1957 [32], and the test was performed with commercial diagnostic kits obtained from Biomedical Diagnostic, Giza, Egypt. To determine AST activity in serum, 0.1 mL of serum samples were mixed with 1 mL of freshly prepared phosphate buffer solution 80 mM pH 6.7 containing 200 mM L-aspartate, lactic dehydrogenase 1.2 IU/mL, maleate dehydrogenase 0.6 IU/mL, NADH 0.18 mM, and a oxoglutarate 12 mM. The absorbance change was recorded at 340 nm for 3 min. AST activity was expressed as U/L, which was calculated by multiplying the absorbance change per minute (ΔA/min) by 1746. Similarly, ALT activity was measured in serum by adding 01 mL of serum to buffer containing L-alanine 560 mM, lactate dehydrogenase 1500 U/L, NADH 0.24 mM, and a oxoglutarate 16 mM. The absorbance change was recorded at 340 nm for 3 min. ALT activity was expressed as U/L and calculated according to the following equation: ALT activity (IU/L) = 1746 × (ΔA/min).

Furthermore, alkaline phosphatase (ALP) activity was measured in the serum samples with the spectrophotometric method described by [33]; a 20 μL serum sample was added to a reaction mixture containing 2-amino 2-methyl 1 propanol pH 10.3, MgCl_2_ 2 mM and 4 -nitrophenylphosphate 50 mM. Then, the absorbance was recorded for 3 min, and ALP activity was presented as U/L with the following formula: ALP U/L = 5454 × (ΔA/min).

#### 2.6.2. Kidney Function

The serum samples were used to determine the renal function. The serum concentration of urea, uric acid, and creatinine were measured. Urea concentration in serum was determined with the diagnostic kits according to the urease-enzymatic method described by [34]. Uric acid was determined in serum samples through the application of uricase–peroxidase coupled reaction [35].Uricase converts uric acid to carbon dioxide and hydrogen peroxide, which react with amino antipyrin in the presence of peroxidase to form a red-color complex. The developed color was measured at 505 nm and with standard uric-acid solution. Creatinine contents in serum are a biochemical marker for kidney function. Creatinine concentrations in collected serum samples were determined according to the methods described by [36].

### 2.7. Serum Antioxidant Enzyme Assay

Serum SOD activity was determined in the prepared serum samples with the colorimetric method described by [37]. SOD activity was measured by determining the ability of the enzyme to inhibit the phenazine methosulphate-mediated reduction of nitroblue tetrazolium dye. This measurement was performed with SOD determination kits purchased from Biodiagnostic, Giza, Egypt.

Serum GST activity was determined according to the method described by [38]. Serum sample (4 μL) was mixed with 980 μL phosphate buffer saline, 10 μL reduced glutathion 100 mM, and 10 μL 1-chloro 2,4-dinitrobenzen 100 mM. The absorbance was recorded every 30 s for 5 min, and GST activity was expressed as μmole/mL/min using extinction coefficient of 1-chloro 2,4-dinitrobenzen 9.6 mM^−1^·cm^−1^.

GSH-Px activity was determined in the prepared serum samples with commercial kits from Biodiagnostic and according to the method described by [39]. The reaction mixture was prepared by adding 10 μL of serum sample to 1 mL of phosphate 50 mM pH = 7 containing 0.1% triton X—100, 0.1 mL of a reagent containing NADPH (2.4 μmol), glutathione reductase (12U), and NADPH (μM). The reaction mixture was added to 100 μL H2O2, and then, the absorbance was recorded at 340 nm for 3 min. GSH-PX activity was calculated as NADPH consumed (nmol/min/mL) with the following equation:

GSH-PX activity (nmol NADPH/min/mL) = (ΔA/min)/0.00622

### 2.8. Molecular Docking of Glutathione Peroxidase

The molecular docking was performed with autodock4 [40]. Glutathione peroxidase structure 5I71 [41] was obtained from a protein data bank. A 40 × 34 × 40 A grid box with −35.57 × 11.83 × 1.54 and with grid-point spacing of 0.375 A was employed. The ligands’ structure was drawn with the PubChem draw-structure tool https://pubchem.ncbi.nlm.nih.gov (accessed on 15 January 2022). The default parameter set of autodock4 was used to generate 10 docking poses. The pose with the best energy score was selected as the most representative.

### 2.9. Statistical Analysis

Results were expressed as mean ± SE of six replicates. For comparing multiple results at once, the recorded data were statistically analyzed by one-way analysis of variance with SPSS version 20.0 for Windows. Mean values were compared with the Least Significant Difference test (LSD) at *p* < 0.05.

## 3. Results

### 3.1. GB Mitigates Lipid Peroxidation and GSH-R in Pb-Exposed Albino Rats

**Figure 1:**
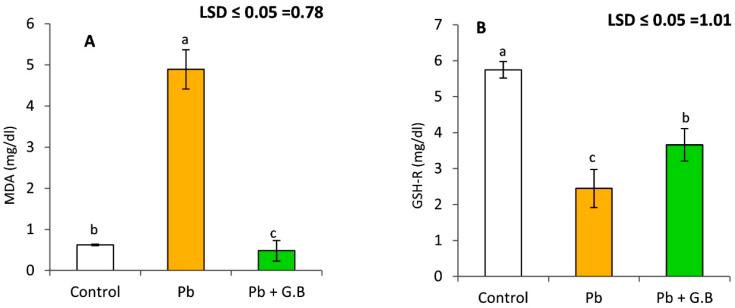
Glycine betaine inhibits Pb-induced lipid peroxidation and leads to reduced glutathione levels in albino rats. (**A**) Lipid peroxidation is expressed as malondialdehyde concentration in serum after 6 weeks of lead-acetate ingestion with or without glycine betaine. (**B**) Serum with reduced glutathione levels after 6 weeks of lead acetate administration in the presence or absence of glycine betaine. Data expressed as the mean of six replicates ± SE. Bars with a different letter are significantly different (*p* < 0.05) as assessed by the LSD test.

Severe tissue damage was evident after 6 weeks of Pb-acetate treatment, as indicated by a highly significant increase in lipid-peroxidation levels, as shown in Figure 1. The results show that the serum MDA concentration in Pb-intoxicated rats was 7.9 times of that in untreated control rats. Supplementation of GB (250 mg/kg bw) to Pb-intoxicated rats mitigated oxidative stress. These results indicate a 70% reduction in lipid peroxidation levels after GB treatment. Moreover, the GSH-R level was significantly reduced by 57.4% with Pb-acetate treatment. GB supplementation with Pb-acetate treatment significantly increased the GSH levels.

### 3.2. GB Protects the Liver against Pb-Induced Dysfunction

**Figure 2:**
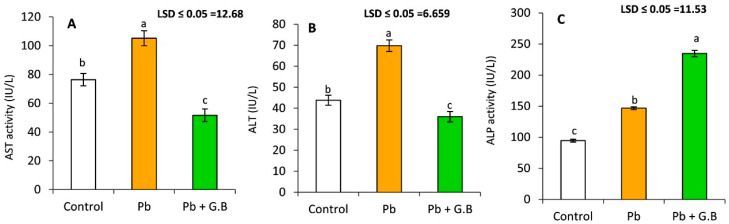
Liver enzymes activities after 6 weeks of exposure to lead acetate (50 mg/kg bw) or lead acetate (50 mg/kg bw) with glycine betaine (250 mg/kg bw). (**A**) Serum aspartate transaminase, AST (IU/L); (**B**) serum alanine transaminase, ALT (IU/L); and (**C**) serum alkaline phosphates, ALP (IU/L). Data expressed as the mean of six replicates ± SE. Bars with a different letter are significantly (*p* < 0.05) different as assessed by the LSD.

The liver is the target organ of Pb-acetate-induced toxicity. As shown in Figure 2, significant increases were observed in the serum activities of AST, ALT, and ALP after 6 weeks of treatment with 50 mg Pb-acetate/kg bw of Pb-acetate by 37.6%, 59.3%, and 55.1%, respectively. GB exerted a reductive effect on both AST and ALT activities.

### 3.3. GB Improves the Kidney Function of Pb-Intoxicated Rats

**Figure 3:**
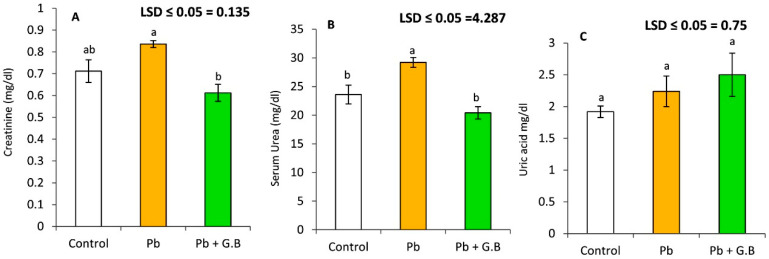
Serum biomarkers of renal function after 6 weeks of exposure to lead acetate (50 mg/kg bw) or lead acetate (50 mg/kg bw) with glycine betaine (250 mg/kg bw). (**A**) Serum creatinine level (mg/dL), (**B**) serum urea concentration (mg/dL), and (**C**) serum uric acid (mg/dL). Data expressed as the mean of six replicates ± SE. Bars with a different letter are significantly different (*p* < 0.05) as assessed by the LSD test.

The kidney regulates fluid composition and volume in the human body. Pb administration resulted in renal dysfunction. Figure 3 clearly shows that Pb-acetate administration (50 mg/kg bw) resulted in significant increases in serum creatinine and urea levels, whereas serum uric acid levels showed a nonsignificant change. Furthermore, GB administration (250 mg/kg bw) following Pb-acetate treatment caused a significant reduction in creatinine and urea levels and a significant elevation in serum uric-acid levels.

### 3.4. GB Enhances the Activity of Antioxidant Defense Enzymes in Pb-Intoxicated Rats

**Figure 4:**
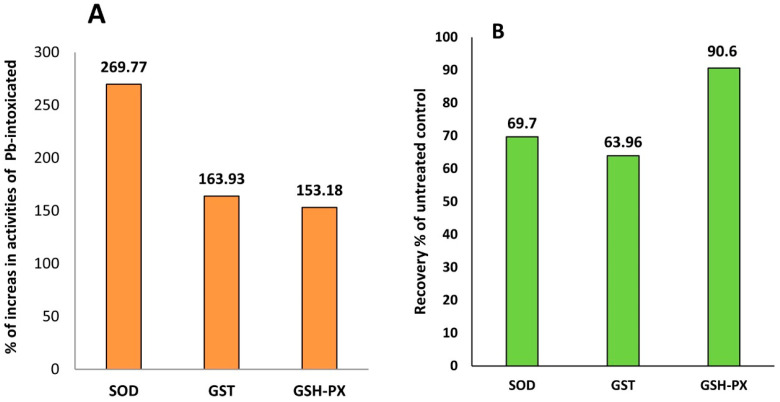
Glycinebetaine improves antioxidant-defense systems in Pb-intoxicated rats: (**A**) % of antioxidant enzyme activities and reduced glutathione levels in Pb-intoxicated rats. (**B**) % of recovery in untreated control rats. Data expressed as the mean of six replicates ± SE. Bars with a different letter are significantly different (*p* < 0.05) as assessed by the LSD test.

As shown in Table 1, the serum activities of SOD, GSH-PX, and GST were significantly decreased in Pb-intoxicated rats compared to control rats. However, the MDA concentration in the serum was significantly increased in rats treated with Pb-acetate. Interestingly, the activities of SOD, GSH-PX, GST, and GSH-R in blood were increased dramatically in Pb-intoxicated rats supplemented with GB by 269.77%, 153.18%, 163.93%, and 149.55%, respectively, as shown in Figure 4. Additionally, GB supplementation with Pb-acetate treatment resulted in a remarkable recovery in the activities of SOD, GST, and GSH-PX by 69.7%, 63.9%, and 90.6%, respectively, as shown in Figure 4.

### 3.5. Molecular Interaction between GB and GSH-PX

**Figure 5:**
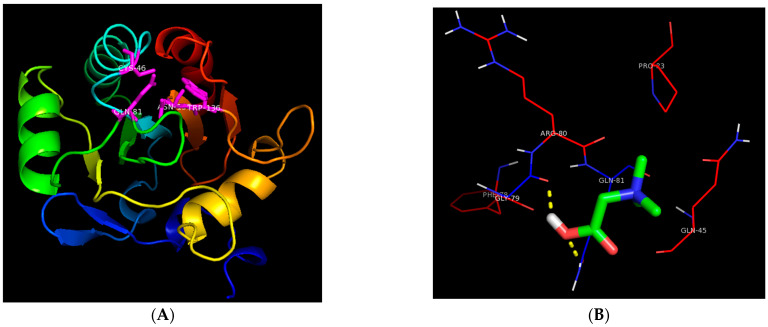
Molecular interaction between glutathione peroxidase catalytic site and GB. (**A**) The molecular structure of glutathione peroxidase 4 from the mouse shown as a cartoon and the catalytic shown as magenta sticks. (**B**) Interaction between glycine betaine and the catalytic site of glutathione peroxidase4, ligand in sticks, residues with polar interaction with ligands in blue lines, hydrogen bonds in yellow dash, and residues with hydrophobic interaction with ligands in red lines. The structure of glutathione peroxidase 4 was cited from [41].

To understand how GB mitigates the inhibitory effect of Pb on GSH-PX, molecular docking was carried out with autodock 4 software. The molecular structure of GSH-PX was illustrated in Figure 5A, including the catalytic site of GSH-PX IV 5I71 Sec46, Gln81, Trp136, and Asn137 as described by [41]. The molecular docking was performed between the catalytic sites of GSH-PX and GB, and H_2_O_2_. The results presented in Figure 5B clearly indicate the presence of a polar interaction between GB and both the amino-acid residue of Gln81 in the catalytic site of GSH-PX and the residue of Gly79 adjacent to the catalytic site. The substrate of GSH-PX, H_2_O_2_ showed higher affinity toward the catalytic site of GSH-PX than GB.

## 4. Discussion

**Figure 6:**
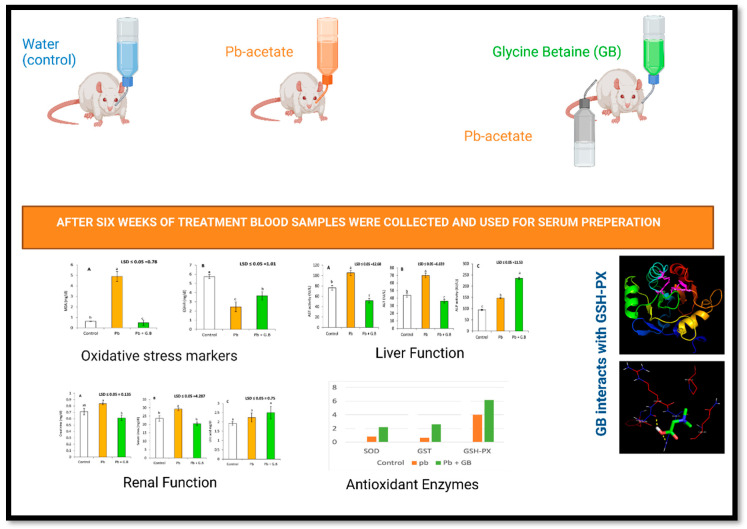
Schematic model for the effect of GB administration on the Pb-induced oxidative toxicity in rats. GB treatment reduced lipid peroxidation and liver enzymes such as AST and ALT in serum, serum urea, and creatinine, and this treatment increased ALP activity, uric acid, and antioxidant enzymes’ activities in serum (SOD, GST, and GSH-PX). GB interacts with the binding site of GSH-PX, which may result in enzyme activation and the mitigation of Pb-induced toxicity.

Several studies demonstrated that Pb-induced oxidative injury could be the main reason for Pb-induced renal and hepatic toxicity [8,10,11,13,42]. The oxidative stress marker, MDA, in liver and kidney homogenates are dramatically increased in Pb-intoxicated rats [43]. Pb treatment resulted in a significant increase (*p* < 0.05) in lipid peroxidation as measured by the levels of MDA as well as a remarkable reduction (*p* < 0.05) in reduced glutathione levels in liver and kidney homogenates [43]. Furthermore, this study demonstrated that Pb ingestion was accompanied by increased serum levels of lipid peroxidation, hepatic enzyme activities, creatinine, urea, and uric acid, and the inhibition of antioxidant defense systems, suggesting that oxidative injury could be a mechanism responsible for Pb-induced hepatic and renal toxicity. Oxidative stress occurs when ROS generation exceeds the capacity of antioxidant defense systems, including antioxidant enzymes [44]. Glutathione is tripeptide-containing cysteine and occurs in reduced and oxidized forms. GSH-R can either function as a cofactor or coenzyme of the enzyme GSH-PX or function nonenzymatically through its ability to scavenge ROS through interaction between ROS and -SH group [45]. The results demonstrated a decline in glutathione levels in Pb-acetate-treated rats. These findings could be attributed to the high affinity of Pb to bind with the -SH groups of GSH-R, a process resulting in oxidative stress (Figure 6).

Interestingly, GB significantly increased GSH-R and decreased the MDA contents in rats treated with Pb-acetate [46]. The observed reduction in MDA suggests the high potential of GB to reduce Pb-induced oxidative stress by increasing GSH-R level and decreasing MDA content in Pb-treated rats. Moreover, the higher levels of MDA, AST, ALT, ALP, creatinine, urea, and uric acid confirmed that Pb could induce oxidative damage in the liver, kidney, and other organs [47,48,49].

The generation of ROS in the case of Pb-induced toxicity occurs through one of the following mechanisms: (i) inhibition of δ-ALAD by Pb and accumulation of the substrate δ-ALA, which can be oxidized to produce ROS [15,43], and (ii) stimulation of ferrous ion-induced membrane lipid peroxidation due to Pb treatment [50]. Consistent with recent studies [51,52], the results of this study confirmed a remarkable inhibition of the antioxidant enzymes (SOD, GSH-PX, and GST) in rats after 6 weeks of Pb-acetate treatment. The inhibition of the activities of antioxidant enzymes could be due to the direct inhibition of antioxidant-defense enzymes through the direct interaction between antioxidant enzymes and Pb ion or through the downregulation of these enzyme genes’ expression. On the other hand, the administration of GB resulted in a remarkable activation of the antioxidant-defense enzyme. The recovery of the activities of antioxidant enzymes reached 90% of the untreated control group in the case of GSH-PX. To understand the effect of GB on the GSH-PX in Pb-intoxicated rats, molecular docking between GB and GSH-PX was carried out. The results of the molecular interaction suggested that the observed activation of GSH-PX was not through binding to the catalytic site. The affinity of GB to bind to the catalytic site of GSH-PX is lower than that of H_2_O_2_. Molecular interaction between GB and the binding site of GSH-PX could not be performed due to the lack of the amino-acid sequence of the GSH-PX binding site in the available published X-ray crystallography structures of GSH-PX.

Several studies have reported that GB exhibits antioxidant [53,54,55] and anti-inflammatory [56,57] activities. GB administration decreased homocysteine levels [57,58] in several organisms and increased S-adenosyl methionine and glutathione contents, with high antioxidant activity [56,57]. The antioxidant potency of GB highly correlates with its effect on the metabolism of sulfur-containing substances such as glutathione in the liver [59]. Although GB does not directly interact with ROS, SAM can scavenge ROS and chelate iron ions that lead to a reduction in hydroxyl radical generation [60]. The present study indicates that GB administration resulted in a remarkable activation of antioxidant-defense enzymes. The observed activation of the antioxidant system could be due to the up regulation of antioxidant-defense genes.

Furthermore, treatment with GB prevents the hepatic injury induced by several toxic substances. For instance, GB administration was found to reduce the accumulation of fats in the liver caused by ethanol consumption [53,56] and high-calorie diets [61]. Moreover, GB treatment was suggested to exert reductive effects on hepatic dysfunction and fibrosis induced by ethanol and carbon tetrachloride [57] and dimethylnitrosamine [59] in rats and by carbon tetrachloride in chicken [55].

## 5. Conclusions

In conclusion, treatment of Pb-intoxicated rats with GB (250 mg/kg body weight/day) for 6 weeks clearly resulted in significant protection of liver and kidney against Pb-induced toxicity through the activation of antioxidant-defense enzymes and the reduction in Pb-induced hepatic and renal toxicity. The molecular interaction between GSH-PX and GB shows that the affinity of GB toward bind to the catalytic site of the enzyme is lower than the affinity of the substrate.

## Figures and Tables

**Table 1:** Recovery of antioxidant-defense enzymes by daily ingestion of GB (250 mg/kg bw) in Pb-intoxicated albino rats. Data expressed as the mean of six replicates ± SE. Bars with a different letter are significantly (*p* < 0.05) different as assessed by the LSD test.

Treatment	SOD	GSTs	GSH-Px
G1 (Control)	3.147 ± 0.23 ^a^	4.074 ± 0.16 ^a^	6.794 ± 0.19 ^a^
G2 (lead acetate)	0.814 ± 0.03 ^d^	0.610 ± 0.01 ^d^	4.020 ± 0.71 ^b^
G3 (lead acetate + GB)	2.196 ± 0.09 ^b^	2.606 ± 0.27 ^b^	6.158 ± 0.07 ^a^

## Data Availability

Not applicable.
